# Indents

**Published:** 1919-03

**Authors:** 


### INDENTS

**☞Brief Articles of Technical or Scientific Value will receive notice and comment. Contributions invited.**

---

RESPIRATORY DISEASES. Col. Victor C. Vaughn, in an address to the American Public Health Association says: So far as the prevention of respiratory diseases is concerned, we do not know much more than our ancestors knew a hundred years ago, and we may as well admit it. I say this in the face of the greatest pestilence that ever struck our country. We are about as ignorant as the Florentines were with the plague described in history. Now that the world war is over and we have whipped the Hun, it devolves upon the medical profession to work hard over the respiratory diseases, and see what we can do for them, and we should not stop until something of great value is discovered.

---

EVILS OF SPIT AND SPITTING IN SPREADING DISEASES. Taliaferro outlines what he terms a "catechism" on "spray borne diseases," and suggests that school children be taught thoroughly on this subject on the basis: "Teach the child and you teach the whole family." Taliaferro claims that 386,030 deaths occur annually from spray borne diseases.—Dr. Taliaferro, in *Southern Medical Journal* for January, 1919.

---

FRACTURES OF LOWER JAW. Imbert and Réal have charge of a center for reconstruction of the face and jaws, and they have had twenty wax casts made of the main types of fracture of the lower jaw in their more than 1,000 cases. These casts have been deposited in the military museum at Paris, but they give photographs of them here with the type of prosthesis required for each. Some of the fractures are like those observed in peace.—*Journal A. M. A.*, Feb. 22, 1919.

CONSOLIDATION OF HEALTH BODIES. A bill has been prepared for presentation before the coming legislature which will provide for the consolidation of all state health organizations under the supervision of a director of public health. Oregon has no less than eleven separate and independent agencies engaged in work pertaining to public health. There are seven special examining boards for various professions. Under the direction of one public official it is believed an up-to-date health administration could be obtained with a maximum protection of health of the people. Under this plan institutions supported by the state would be at the disposal of a health director, for use by organized societies. Thus the laboratories of the University of Oregon Medical School could be used in conjunction with the laboratory of the state board of health. The working out of this proposed plan will be observed with much interest.—*Northwest Medicine.*

---

NICKEL-COPPER AMALGAM. Nickel-copper amalgam, in the opinion of those who are acquainted with it, has all the virtues of copper amalgam without any of its disadvantages. I have used it with every satisfaction during the last three years or more, many of the fillings being constantly under observation. In my own case I have had a large approximo-occlusal nickel-copper amalgam filling in a second molar for a considerable time, and it stands the stress of mastication perfectly, comparing very favorably with a high-grade similar filling in the tooth adjoining. It keeps a good color and does not stain the dentine, nor does it show any signs of cupping.—S. Lever, *Commonwealth Dental Review.*

---

SEPARATING TAPE. Soak linen tape in a thin solution of gutta-percha in chloroform until it becomes saturated. The chloroform then evaporates and leaves a gutta-percha tape, the toughest and most effective material that has ever been suggested for separation. It does not disintegrate like plain linen; it remains perfectly in place and does not irritate the soft tissues. Its action, while very effectual, is so gradual that the patients seldom or never complain of discomfort.—*Dental Review.*

---

PULP PROTECTION. These days of pulp conservation demand pulp protection under large metallic or synthetic restorations. Oxyphosphate of zinc, and even the chloride of zinc frequently cause irritation and death to the pulp. Also the bacterial invaded dentin overlying this portion of the pulp is a menace to its life unless an effort is made to give the pulp protection. A paste made of equal parts of hydronapthtol and zinc oxide with a drop or two of equal parts of oil of cloves and camphophenique, should be carefully spread over the wall of dentine closest to the pulp and then sealed in with oxyphosphate of zinc.—F. W. Frahm in *Pacific Gazette.*

---

BARBED BROACHES FOR MOLARS. The ordinary barbed broach has too long a shank for use in molars and some bicuspids. A very useful barbed broach can be made by cutting the shank as short as desirable for the operation and then sealing a bulbous handle on the upper end of the remaining shank with hot sealing wax. This instrument has a very delicate touch.—F. W. Frahm in *Pacific Gazette.*

---

SEALING ARSENIC IN A TOOTH. I wish to present a method of sealing in arsenical paste that may interest Army dental officers. A country dentist has much the same troubles as the Army dentist. Farmer patients often remain away for weeks, or even months, after having a ''tooth-ache'' stopped by the application of arsenic. To aviod danger of leaking, I have been sealing the treatment in with soft amalgam. Use a soft tin amalgam mixed with an excess of mercury. It is not necessary to prepare the cavity, except to remove the soft decay. If the cavity should be in a position where the arsenic could not be sealed in safely, fill with gutta-percha, and then make a small cavity in sound dentin, sealing in the arsenic with soft amalgam.—Dr. Heckert in *Items of Interest,* January, 1919.

---

OUR NEXT DUTY. Are we giving our best efforts to the country, or do we think that because the war is over we can take life easy? Prosperity is up to us! Keep the wheels turning! We must all do our best to make the change from war work to peace work as easy as possible. Co-operation is the big thing needed *now.*—U. S. Dept. of Labor, Wm. B. Wilson, Secretary.

---

POLISHING GROUND PORCELAIN. Make a very soft paste of a saturated solution of gum camphor and pumice; place in spirit of turpentine. Keep the polishing wheel wet with this while polishing the ground surface.—W. H. Spaulding, *Dental Summary.*

---

REPAIRS IN PINK VULCANITE. The discoloration always attending the union of new and old vulcanite can be removed by tracing a little nitric or hydrochloric acid over the discoloration. In a few seconds it should be neutralized with sodium bicarbonate or ammonia. The vulcanite will not suffer any damage if care is exercised. —F. W. Frahm, in *Pacific Gazette.*

---

FATAL COCAIN POISONING. A man of 52 died on the table after local anesthesia for a herniotomy, and it was found that a 10 per cent. solution of cocain had been injected by mistake instead of the supposed 2 per cent. novocain solution. The bottles were kept on the same shelf, with the same size, shape and color of label.

Moller protests against this practice, urging that dangerous substances should be kept in special vials. The sudden death had been inexplicable at first, the convulsions, clonic spasms, cyanosis and coma proving fatal notwithstanding stimulants, artificial respiration and massage of the heart. This communication is an abstract from a Swedish journal.

---

"A Man's a Man for a' That." Do you think of your Italian acquaintance as a "Dago"? Marconi is of the same race. Do you refer to your Polish neighbor as a "Polak"? Paderewski is a Pole. Are your Scandinavian fellow-workers "square-heads" in your mind? The inventor of dynamite and the armored battleship were men of that stock. And this argument applies to every race that has found a home in America. It is wrong thinking to use slurring names, even in your mind, about the men of another race. Think straight, and judge a man by his character, not by his birthplace.

---

"While We Sleep." The rights and property of the Bayer Company, together with its subsidiaries, was recently sold at auction by the alien-property custodian, Mr. A. Mitchell Palmer. This German-owned chemical manufacturing company, the best-known product of which is aspirin, did a business, in 1917, amounting to $5,608,502.51, with net profits of $1,768,566.78. It was sold to the Sterling Products Company, of Wheeling, West Virginia, a "patent-medicine" concern, for $5,310,000.

It is interesting and significant that aspirin (of the Bayer cross) and Cascarets now have a common owner. Can it be possible that the Sterling Products Company picked up the five plus million dollars, required for effecting this purchase, from the laxative tablet that "works while you sleep?"

Hot upon the trail of the preceding item of news comes the statement that the patent-office has cancelled the Bayer-owned trademark of the word "Aspirin."—*Clinical Medicine.*

---

PROGRESS OR RETROGRESSION. In one of the leading medical journals of the country, the following two items were found, which deserve mention. They are as follows:

> Argument never settled anything. How calm and collected is the man who has the facts! I "think" doesn't weigh much.

> A wise old man was Oliver Ding,
> By few words was he moved,
> He never would believe a thing
> Until it had been proved.

According to this mode of reasoning, it would be futile to attempt any investigation or to deal with any problem whatever "until it has been proved," that is to say, until it has been *definitely* settled. It would be exactly like forbidding Tommy to go into the water until he has learned to swim.

The absurdity of such an attitude is self-evident and, according to its logical conclusion, it precludes any investigation, study, and endeavor looking to progress or improvement. It was the accusers and judges of Galileo that, like the wise old man, Oliver Ding, would not believe a thing that had not been absolutely proved. Since, to their own limited minds, the learned Galileo could not offer the proof that the earth journeys around the sun, isstead of the reverse, Galileo was forced to recant and to do penance for daring to believe things that had not been "proved." Similarly, the medical leaders of his day refused to accept Harvey's demonstration of the circulation of the blood, denying that it had been proved. The conclusions drawn by Edward Jenner from the results of his observation after inoculating the boy, James Phipps, from material obtained from cow-pox-pustules from the hands of the dairy-maid, Sarah Nelmes, were fiercely combated by the medical leaders of the day, who refused to believe a thing until it had been "proved;" and the same fate was accorded to Servetus, Semmelweis, Holmes, Pasteur, Lister, Koch, and all the rest of "innovators" that dared to strike out independently along new lines of thought and in new directions of practice.

We submit that the man who refuses to consider possibilities, even though they be not definitely proved, is one of those that remain in a rut all their lives, who are afraid to progress lest they lose themselves in the deep waters of unorthodox teachings; who fear to think independently, preferring the easier and less onerous, if less glorious, part of those that follow well-established leaders.

For our part, while we are not exactly constantly eager for new things, in the manner of the Athenians of old, we can not believe that our own wonderful era has attained and possesses all that is worth knowing. There is so much to be learned, so much to be discovered, that we prefer to maintain an open mind for new things that may be presented to our understanding; tentatively, perhaps, and, before they have been proved. We fully appreciate, that there are many facts that are matters of course today, but were strange, unproved, and even improbable or impossible but on the yesterday.

The sentiments expressed in the two items introducing this editorial, are reactionary in their tendency and injurious to every hope of progress. It is to be deplored that they should have found an approved place in a prominent and leading medical journal.—*Clinical Medicine.*

---

BLEACHING A TOOTH. Pyrozone (25 per cent. hydrogen peroxide.) To apply: Be sure that apex is sealed. Place dry cotton in open part of canal and in pulp chamber; seal with temporary stopping; with a hot plugger drive a small hole through center of stopping to cotton. Take tube of pyrozone (which is extremely volatile), hold in towel saturated with ice water and break off tip of tube with plyers. Take long thin operating plyers, and carry liquid between closed beaks through hole in stopping to dry cotton in canal. Repeat several times until cotton is saturated. Now seal hole in stopping. Usually one application of pyrozone, if this procedure is followed out accurately, will give a very satisfactory result, though it is sometimes necessary to repeat it once or twice. It is often well to enlarge the cavity or canal from the lingual, lessening the thickness of the discolored dentine to be penetrated by the bleach. If stain is due to metal such as German silver post or amalgam, this treatment will not benefit much.—V. C. Smedley, in *Digest.*

---

DENTAL WAR SOUVENIR. I am sending herewith what I might call a souvenir of the battle of Cambria—which I thought would be of special interest to you. This aluminum denture was found beside a dead German Machine Gunner, on the field, during our advance. He had evidently been blown up by a shell, as the denture had fallen out of his mouth. It is only of interest as showing German methods in Army Dentistry, and although a little crude, it strikes me, judging from the amount of trouble I have with men breaking their dentures or chipping teeth off them with hardtack, that it has many advantages for active service over porcelain teeth and vulcanite.

The piece is cast aluminum, teeth and base being composed entirely of aluminum, apparently made from one casting, with clasps of apparently a gold alloy imbedded in the substance of the casting. It also has the appearance of having been duplicated from a previously made vulcanite denture of the ordinary type.—Captain Grant, in *Oral Health.*

---

SETTING CROWNS AND BRIDGES. Any crown or bridge may be firmly set, and still be removed at a later date without destroying either the piece of work or the supporting tooth or root. After the crown (esthetic) has been polished and is ready to set, the pin and plate is coated with one or two coats of chloropercha, allowing each coat to dry before the next one is applied. Then try the crown in place on the root to make sure it can be seated. It is now cemented to place in the usual manner. Gold contour crowns, such as the shell crown, must have all inside undercuts filled up with guttapercha, then warmed and placed over the tooth stump. After trying it in place several times to test its articulation it may be cemented in the usual manner. The Orton and shoulder crown is given a coat or two of the chloropercha on the walls that will be in contact with the axial and occlusal walls of the tooth stump. They are then tested and cemented to place in the usual manner. When crowns and bridges have thus been prepared they can be removed at a later date by simply heating them to soften the chloro and guttapercha and applying traction.—F. W. Frahm, in *Pacific Gazette.*

---

An Easy Method of Applying Iodin. The tincture of iodin, either in the form of Talbot's iodoglycerol solution for various forms of gingival inflammation or the disclosing solution in prophylactic work, or in combination with aconitecapsicum as a counter-irritant in peristeal disturbances, can best be applied with the aid of wooden applicators. These may be procured from any druggist in the form of wooden sticks about 12 inches long and 1½ mm. in diameter. A small whisp of cotton twisted around the end of the applicator enables one to apply the iodin without danger of corrosion of steel instruments. The end of the applicator is broken off and discarded as used. This I have found to be a convenient and clean method of using a drug which has a tendency to spread over the mouth.—W. H. Savage, *Dental Cosmos.*

